# Rectal Cancer Presenting with Absceding Infection Due to *Fusobacterium nucleatum*

**DOI:** 10.3390/pathogens11101113

**Published:** 2022-09-28

**Authors:** Sebastian Zundler, Christian Mardin, Simone Bertz, Francesco Vitali, Richard Strauß, Julia Fürst, Markus F. Neurath, Deike Strobel

**Affiliations:** 1 Department of Medicine 1, University Hospital Erlangen, Friedrich-Alexander-Universität Erlangen-Nürnberg, 91054 Erlangen, Germany; 2 Department of Ophthalmology, University Hospital Erlangen, Friedrich-Alexander-Universität Erlangen-Nürnberg, 91054 Erlangen, Germany; 3 Institute of Pathology, University Hospital Erlangen, Friedrich-Alexander-Universität Erlangen-Nürnberg, 91054 Erlangen, Germany

**Keywords:** rectal cancer, *Fusobacterium nucleatum*, liver abscess, endophthalmitis

## Abstract

Intestinal microbiota such as *Fusobacterium nucleatum* play an important role in the pathogenesis of colorectal cancer. Here, we describe the case of a 47-year-old patient presenting with endophthalmitis and a liver abscess due to *Fusobacterium nucleatum* that prompted the diagnosis of colorectal cancer as the most likely source of infection. This case highlights that colorectal cancer needs to be considered in patients with systemic infection with *Fusobacterium nucleatum* and colonoscopy should be performed.

## 1. Introduction

The pathogenesis of colorectal cancer is marked by accumulating mutations in pathways of cell cycle, proliferation and cell death leading to uncontrolled tumor growth [1]. However, the role of the tumor microenvironment for the progression and prognosis of colorectal cancer has recently gained increasing attention [2]. This microenvironment also includes the intestinal microbiota and potentially non-autochthonous bacteria, which have a crucial function for colorectal carcinogenesis [3]. In particular, a negative impact of *Fusobacterium nucleatum* including increased proliferation of colorectal cancer cells [4], the promotion of metastases [5] and resistance to chemotherapy [6] has been demonstrated.

Here, we report the unique case of a patient with systemic infection with *Fusobacterium nucleatum* that resulted in the diagnosis of rectal cancer and facilitated successful therapy.

## 2. Case Report

We describe the case of a 47-year-old male Caucasian patient who presented with progressive loss of vision on the right eye. He also reported abdominal discomfort over the past three weeks as well as fevers and chills during the previous 10 days.

**Figure 1:**
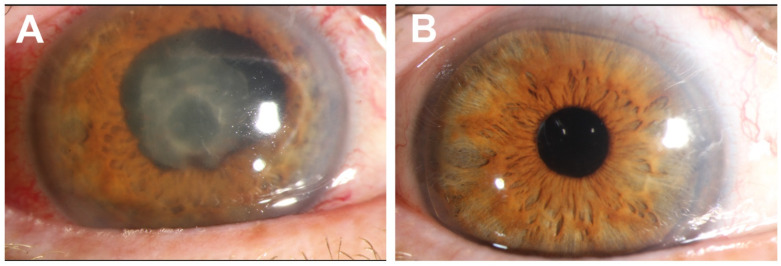
(**A**) Clinical findings of the right eye at initial presentation with corneal edema, hypopyon (covered by the lid), posterior synechia, fibrin on the lens. (**B**) Clinical findings of the right eye 10 weeks after vitrectomy: Irritation-free anterior chamber, centered lens of the posterior chamber, silicon oil in the vitreous space.

Upon admission, the patient had a temperature of 39 °C. Clinical examination revealed a hypopyon of the right eye (Figure 1A). Laboratory tests showed elevated leukocyte counts (20.56 G/L), abnormal liver function tests (GPT 97 U/L) and a C-reactive protein level of 172 mg/L (normal < 5 mg/L). The diagnosis of endophthalmitis was made and immediate vitrectomy was performed. Empiric antibiotic treatment with meropenem and vancomycin was initiated.

**Figure 2:**
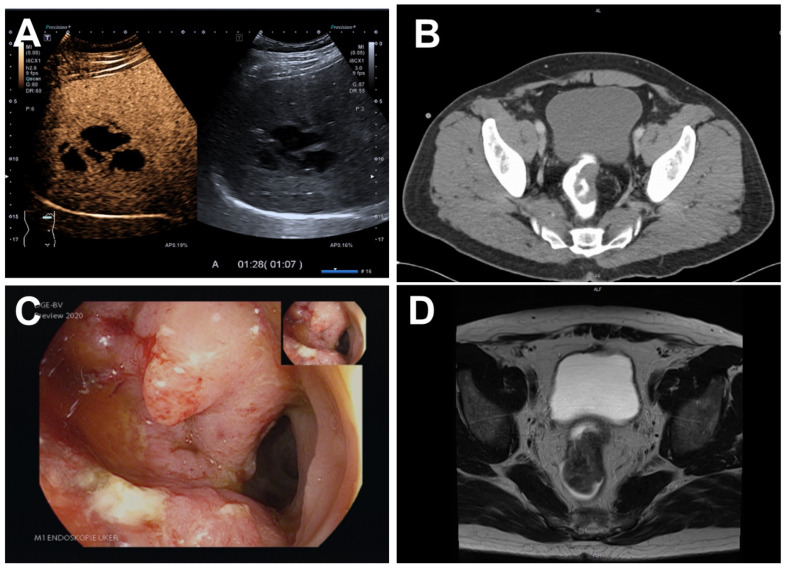
(**A**) Contrast-enhanced ultrasound of the liver abscess in the portal venous phase. (**B**) Initial CT scan revealing a tumor in the rectum. (**C**) Endoscopy of the tumor in the rectum. (**D**) Initial MRI scan of the tumor in the rectum.

Upon referral to diagnostic ultrasound, B-mode sonography and contrast-enhanced ultrasound (Figure 2A) identified an abscess in the right liver lobe. A 10 French pigtail drainage catheter was placed.

No pathogens grew in blood cultures and cultures of the vitreous humor. Real-time PCR of the vitreous humor was negative for *Tropheryma whipplei*, *Staphylococcus aureus, cytomegalovirus*, *herpes simplex* and *varicella zoster* virus DNA. Serologic results for *galactomannan, Candida* antigen, beta-D-glucan, *Borrelia burgdorferi* and *Treponema pallidum* were negative. However, PCR of the vitreous humor and the liver abscess using pan-bacterial primers were positive and *Fusobacterium nucleatum* was exclusively detected by sequence analysis (sequencing performed by eurofins) in both materials. Moreover, *Fusobacterium nucleatum* grew on Schaedler agar with vitamin K and was subsequently detected by mass spectrometry (MALDI-TOF) in cultures of the liver abscess. Since a dental focus had been ruled out a few days prior to hospital admission, these results were overall consistent with systemic *Fusobacterium nucleatum* infection without an obvious port of entry.

Hence, a thoracic/abdominal/pelvic CT scan revealed a tumor in the rectum (Figure 2B) in addition to the known liver abscess in segment 6/7. Consistently, colonoscopy was suspicious for rectal cancer (Figure 2C) and histopathological analysis confirmed a moderately differentiated intestinal-type adenocarcinoma. Staging including MRI of the pelvis characterized the carcinoma as cT3b cN0 (Figure 2D).

The drainage of the liver abscess was stopped after four days. Antibiotic treatment included four weeks of IV meropenem followed by four weeks of an oral sequential therapy with amoxicillin/clavulanic acid. Activity against *Fusobacterium nucleatum* has previously been demonstrated for both agents [7]. Follow-up liver B-mode sonography showed consolidation of the abscess at 10 days and a complete resolution after 6 weeks. Following further ophthalmologic interventions, the patient partially regained vision (Figure 1B). With regards to rectal cancer, he received neoadjuvant radio-chemotherapy, deep anterior rectum resection and adjuvant radiochemotherapy.

## 3. Discussion

Taken together, we consider the rectal cancer as the most likely source of invasive infection with *Fusobacterium nucleatum* in this patient. *Fusobacterium nucleatum* is an oral bacterium rarely found in healthy gut and colorectal cancer is a precondition for its colonization [8]. Several bacterial species have previously been linked to colorectal carcinogenesis [9] and recent studies even suggested a causal role for *Fusobacterium nucleatum* [4,10,11]. Although we were not able to verify the presence of *Fusobacterium nucleatum* in cancer tissue from our patient, we did not find an oral–gingival or other port of entry. Notably, in contrast to vitreous humor and liver abscess material, tissue samples were obtained after several days of antibiotic treatment substantially reducing the probability of successful detection [12].

Thus, to our knowledge, we describe the first case of undiagnosed colorectal cancer presenting primarily with systemic infection with *Fusobacterium nucleatum*. It shows that bacteria involved in the pathogenesis of colorectal cancer may not only play a local role, but also become invasive. This highlights the necessity to consider colorectal cancer in the differential diagnosis of systemic infection with *Fusobacterium nucleatum* and to include endoscopy in the diagnostic work-up, since this might expedite diagnosis and treatment and, thus, improve prognosis.

## Data Availability

Not applicable.
